# Tribological performance of IL/(GO-MWCNT) coatings in high-vacuum and irradiation environments

**DOI:** 10.1038/s41598-022-15914-z

**Published:** 2022-08-23

**Authors:** Lili Zhang, Zhengrui Zhang, Ahad Amini Pishro, Siti Jahara Matlan

**Affiliations:** 1 College of Civil Engineering, Sichuan University of Science & Engineering, Zigong, Sichuan 643000 People’s Republic of China; 2 Civil Engineering Programme, Faculty of Engineering, University Malaysia Sabah, 88400 Kota Kinabalu, Sabah Malaysia

**Keywords:** Physics, Chemical physics

## Abstract

In this paper, we investigated the effect of space irradiation on the lubricating properties of IL/(GO-MWCNT) solid–liquid lubricating coatings. The solid–liquid lubricating coatings consist of ionic liquids (IL), graphene oxide (GO), and multi-walled carbon nanotubes (MWCNT). Irradiation experiments were carried out using ground simulation equipment. Atomic oxygen (AO), ultraviolet (UV), proton (Pr), and electron (El) irradiation alter the composition, structure, morphology, and tribological properties of solid–liquid lubricating coatings. The experimental results show the composition changes induced by irradiation, including the decomposition of ILs lubricants. The damage to the lubricating material was the most serious by Pr irradiation and the least by UV irradiation.

## Introduction

Owing to their wide range of potential benefits, the use of solid–liquid composite lubrication systems^1–4^ in the automotive and aerospace industries has been enthusiastically promoted over the last two decades. The solid–liquid composite lubrication system consists of base fluids and nano additives. Base fluids are mainly used to reduce the friction between the surfaces of the moving parts, while the machine equipment with cooling, sealing, corrosion, rust, insulation, power transmission, cleaning impurities, etc.^5^. Nano additives have the potential to reduce the friction and wear of moving parts and enhance the machine's durability^6–9^.

Numerous studies have examined the effect of adding various nanoparticles to oil-based nano lubricants. Niraj Kumar et al. have been; reported that the anti-wear properties of palm oil are enhanced due to the addition of α and β-MnO_2_ nanorods with diameters of approximately 10–40 nm^10^. According to Jatti et al.^11^, using CuO as an additive enhances mineral-based multigrade engine oil's friction and wear properties. They report that the CuO nano additive converts sliding friction to rolling friction, thereby reducing the effective coefficient of friction between the rubbing surfaces. Vlad Bogdan Nist et al.^12,13^ reported that WS_2_ nanoparticles reacted with the steel substrate under high temperature and pressure to form a protective tribo-film, thereby reducing the penetration of H into rolling element bearings and thus preventing H embrittlement. It has also been reported that composite additives outperform single additives^14^. Arvind Kumar et al.^15^ explored polymer-functionalized graphene-based nanocomposites as lubricant additives, which can replace traditional bulk materials due to their nanoscale size and good mechanical and thermal properties. Ramón-Raygoza et al.^16^ reported enhanced tribological behavior of multilayer graphene impregnated with copper (MLG-Cu). Luo et al.^17^ investigated the lubricating properties of graphene additives with different degrees of exfoliation, providing new insights into the relationship between friction-induced nanostructure evolution and the lubricating properties of graphene as a lubricating additive. This result has excellent potential for the structural design of graphene as a lubricant additive.

Recently, solid–liquid synergistic lubrication based on diamond-like carbon (DLC) coatings has become an attractive lubrication system due to its ultra-low friction and good wear resistance in all lubrication regimes^18,19^. Nickel nanoparticles (average diameter 7 nm) capped with oleylamine and oleic acid were added to the DLC/DIOS solid–liquid synergistic lubrication system^20^. In all lubrication schemes, the lubrication performance of the system was significantly improved by the addition of Ni nanoparticles. The friction coefficient is reduced by 10.3–19.1%, and the wear rate of DLC can be reduced by 50% in the state of boundary lubrication. We previously prepared DLC/IL/(GO-MWCNT) coatings, which exhibited friction-reducing properties under high vacuum conditions. The nanofluids also showed improved wear resistance by transferring graphene and multi-walled carbon nanotubes as separators. Their synergistic effect significantly enhanced the IL-GO/MWCNT composites. However, for the space environment, high vacuum is only one of the conditions, such as space conditions, including high and low temperature (HT/LT), atomic oxygen, UV irradiation, proton and electron beam irradiation, and the absence of a gravitational field^21,22^. In the low-pressure environment, AO is one of the most damaging and dominant neutral species (approximately 80%) in the upper atmospheric from 200 to 700 km. It is well known that atomic oxygen is closely related to the failure of liquid lubricants due to the severe degradation and evaporation under AO irradiation^23^. Studying the effect of other space conditions on DLC-based solid–liquid lubricating coatings is essential.

In this study, we investigate the tribological properties of IL/(GO-MWCNT) coatings before and after simulated space irradiation, including AO, UV, EL, and Pr, to elucidate whether the coatings adapt to the space environment. The composite materials and morphologies of the worn surfaces were systematically analyzed, revealing the friction and wear mechanism.

## Experimental

### Material

ILs 1-butyl-3-methylimidazolium tetrafluoroborate (purity, 97%) was synthesized and provided by the State Key Laboratory of Solid Lubrication, Lanzhou Institute of Chemical Physics. Powders of multilayer GO were purchased from Nanjing XFNANO Materials Tech Co., Ltd. MWCNTs were sectioned as previously described^24^. All the other materials were used as received.

### Preparation of nanofluids and hybrid films

The optimum mass ratio of GO to MWCNTs (30:70) and the total concentration (0.075 mg mL^−1^) obtained by the previous screen test were adopted for the additives in the test^24,25^. The IL 1-butyl-3-methylimidazolium tetrafluoroborate ([BMIM]BF_4_, purity, 97%) was used in this experiment. The dispersions and hybrid films were prepared as previously described^25^. Before each friction test, the ILs containing the additive were sonicated for 15 min to uniformly disperse the carbon nanotubes and graphene, and 5 μL nanofluids on the steel surface were taken with a micro-injector. ILs with a GO: MWCNTs mass ratio of 30:70 was abbreviated as IL-GO30. To check the repeatability of the experiment, each friction test was carried out at least three times under the same conditions.

### Irradiation procedure

Experiments involving Pr, UV, AO, and El irradiation of the hybrid films were performed in ground‐based simulation facilities at the Lanzhou Institute of Chemical Physics, Chinese Academy of Sciences. For the AO irradiation, the average kinetic energy was approximately 5 eV, which is analogous to the energy of AO impacting the surface of a spacecraft in a space environment^26,27^. The flux of AO was approximately 6 × 10^15^ atoms cm^2^ s^−1^, which was measured via the standard method of Kapton mass loss. The exposure time of AO was controlled at approximately 120 min. Using a Hg-Xe lamp, the UV irradiation test was performed under excimer light with a wavelength range of 115–400 nm in a high-vacuum environment (4.0 × 10^−4^ Pa). The typical UV energy flux was six times the solar constant. The exposure time was controlled at 120 min. The Pr and El irradiations were performed at an acceleration voltage of 25 kV under a pressure of 4.0 × 10^−4^ Pa. The fluxes of protons and electrons were approximately 6.25 × 10^15^ and 2.5 × 10^14^ ions cm^2^ s^−1^, respectively. The Pr and El irradiation times were controlled at approximately 10 and 120 min, respectively, because the Pr and El irradiations had higher energy than the AO and UV irradiations. The speed of a spacecraft in outer space is approximately 7 to 8 km/s, and this relative velocity endowed the particles with a flux about 10^12^–10^15^ atoms cm^2^ s^−1^^28,29^. Therefore, in our irradiation device, the flux of Pr, AO, and El irradiation was close to that of AO produced by electron cyclotron resonance microwave plasma technology and had mean kinetic energy of 5 eV, which is similar to the energy of AO impinging on the surface of a spacecraft in a space environment^30^. In addition to the simulated space irradiation, the samples underwent friction and wear tests.

### Characterization of tribological properties

All friction tests were conducted using the same homemade rotational ball-on-disk vacuum tribometer in a high vacuum (10^−4^ Pa). The friction force resolution of the tribometer is 1 μN. Commercially available steel balls (AISI-52100) with a diameter of 3 mm were used as the counterparts. The steel balls were ultrasonically cleaned in acetone for each test. Sliding experiments were performed with a normal pressure of 5 N. Each friction test lasted 60 min, and the friction coefficient was recorded as the average value in the steady-state. The experimental parameters of the simulated space irradiation and friction tests are presented in Table 1.

**Table 1:** Experimental parameters of the simulated space irradiation and friction tests.

Irradiation	Parameters	Friction condition
AO	4.6 × 10^15^atom cm^2^ s^−1^, 5 eV, 2 h	5 N, 300 r/min, 60 min, 4 × 10^−4^ Pa
El	500 μA/cm^2^, 2 h, 25 KV, 2.5 × 10^14^ cm^−2^ s^−1^	5 N, 300 r/min, 60 min, 4× 10^−4^ Pa
UV	700 W/m^2^, 115–400 nm, 2 h	5 N, 300 r/min, 60 min, 4 × 10^−4^ Pa
PR	25 KV, 2.5 × 10^14^ cm^−2^ s^−1^, 10 min	5 N, 300 r/min, 60 min, 4 × 10^−4^ Pa

### Compositional and structural characterization

The graphene and MWCNTs were characterized via high-resolution transmission electron microscopy (HRTEM, JEM-2010). The wear scar diameters on the steel balls were measured using an optical microscope (STM6, Olympus). After friction tests, the wear depth and wear track profiles were determined by a noncontact three-dimensional surface profiler (model Micro MAXTM, ADE Phase Shift, Tucson, AZ). The disk wear rate was calculated using the wear depth^24^. The wear rates given in this paper are the average values from the three replicated tests.

The changes in the chemical composition of the nanofluids were investigated via time-of-flight secondary-ion mass spectroscopy (TOF-SIMS, ION TOF-SIMS IV). After the friction test, the TOF-SIMS test specimens were ultrasonically cleaned with acetone for 10 min^25^.

## Results and discussion

### Tribological characterization

**Figure 1:**
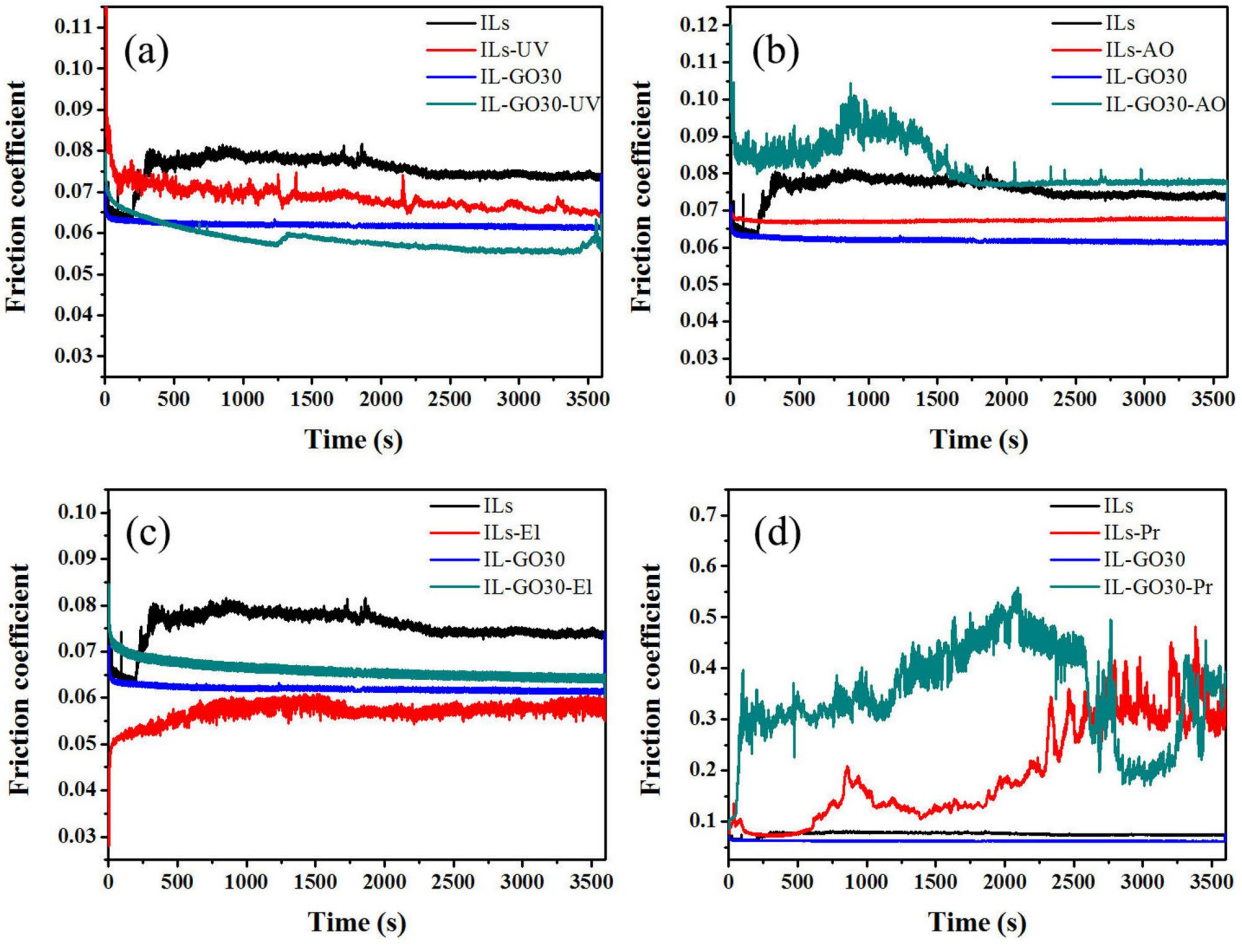
Comparison of the tribological behaviors of the composite coatings of steel/ILs, steel/IL-GO30 sliding against a steel ball under high vacuum condition. ILs and IL-GO30 represent the friction coefficients of the sample before irradiation. ILs-UV (or AO, El, Pr) and IL-GO30-UV (or AO, El, Pr) represent the friction coefficients of the sample after irradiation.

Figure 1 shows the space tribological behaviors of the composite coatings of steel/ILs and steel/IL-GO30 sliding against a steel ball under high-vacuum conditions. The lubricants exhibited different friction and wear behaviors after irradiation. As shown in Fig. 1a, before UV irradiation, the friction coefficient of the lubricant with compound additives is lower than that of the ILs. At the beginning of the friction test, the friction coefficient was low. After 400 s, it increased to almost 0.08. By adding the compound additive, the friction coefficient curve was very smooth. This result demonstrates that the additive can reduce the friction coefficient. The friction coefficients of both ILs and IL-GO30 were smaller than those of the lubricants after UV irradiation. As shown in Fig. 1b, the friction coefficient of IL-GO30 (after AO irradiation) was slightly larger than that of ILs. As shown in Fig. 1c, the effect of the El irradiation on the lubricant was insignificant. Although the friction coefficient of IL-GO30 was slightly larger than that of ILs after El irradiation, the friction coefficient of IL-GO30 was more stable. As shown in Fig. 1d, the Pr irradiation significantly impacted the friction coefficients of ILs and IL-GO30. The friction coefficients of ILs and IL-GO30 fluctuated sharply during the whole friction process after the Pr irradiation. This revealed that ILs and IL-GO30 could not improve the friction-reducing properties effectively when the nanofluids suffered a lubrication failure in the steel/steel frictional pairs under Pr irradiation.

**Figure 2:**
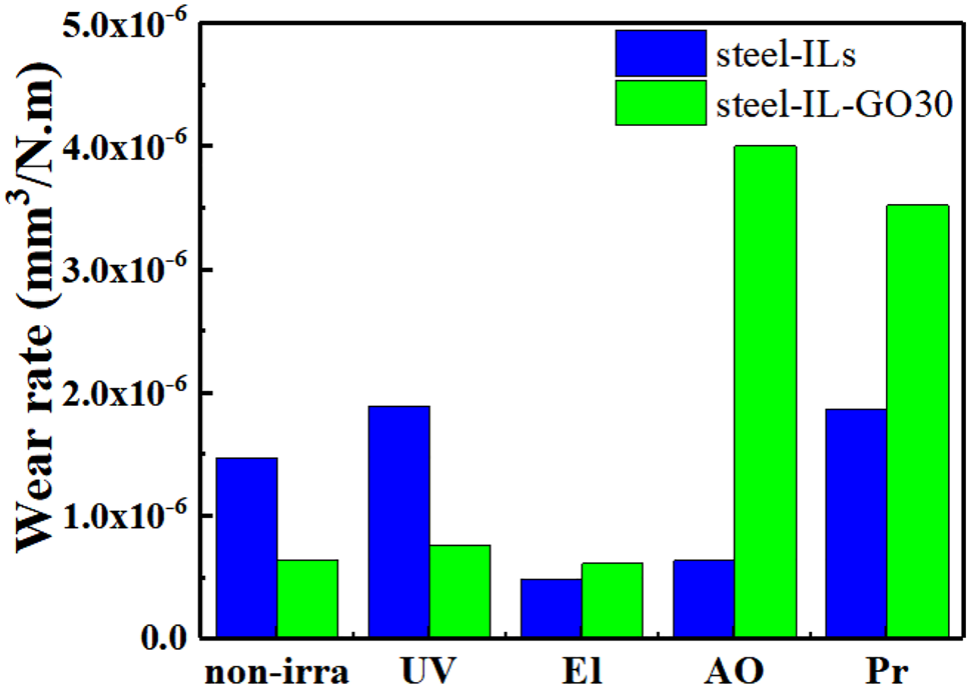
The corresponding disc wear rates.

**Figure 3:**
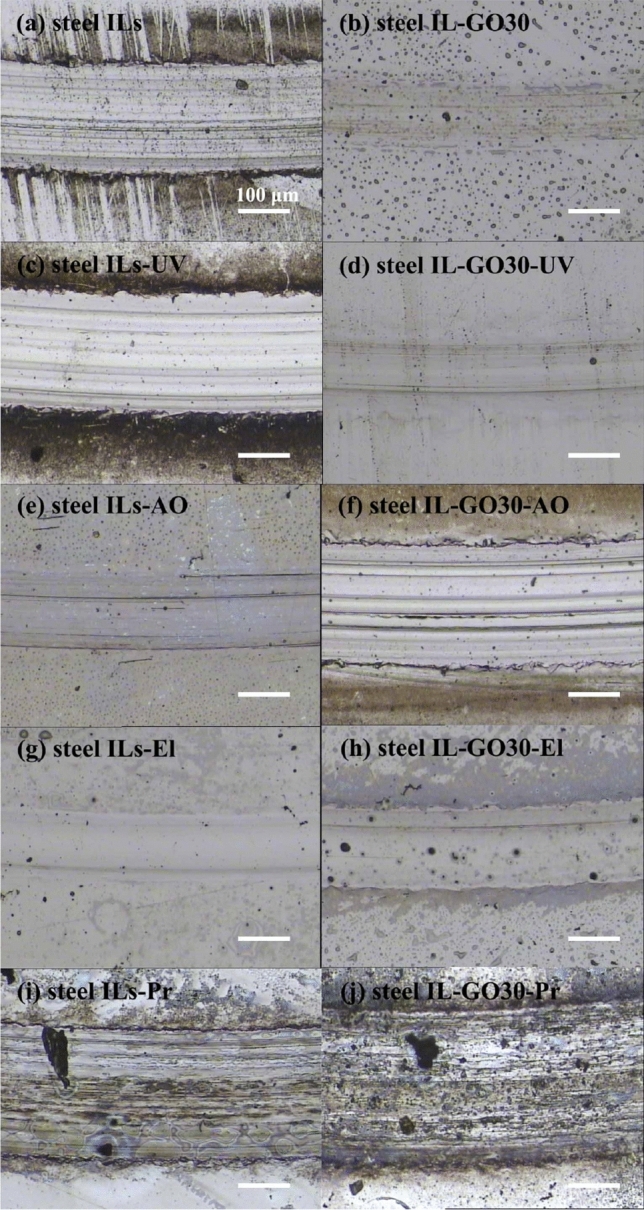
Optical micrographs of the wear scars of steel films before and after irradiations.

**Figure 4:**
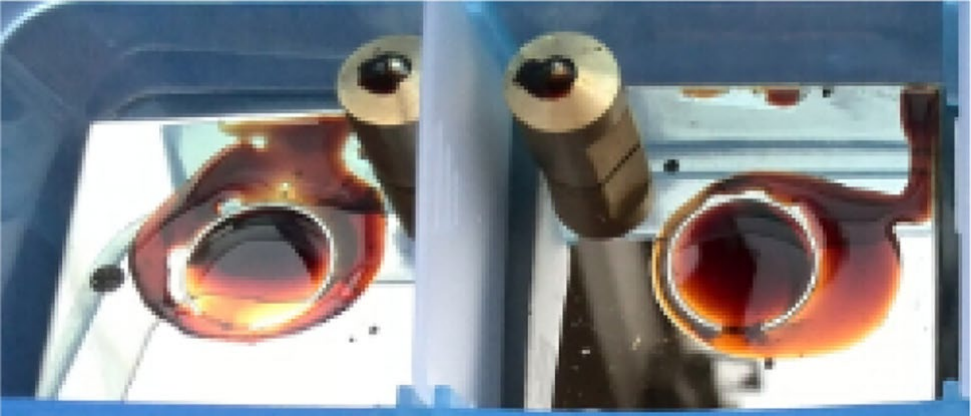
Photographic image of composite films after Pr irradiation and friction test.

Figures 2 and 3 summarize the wear results for the composites coatings under different irradiation compared with those for the case of no-irradiation. Overall, the magnitude of the disc wear rates for these solid–liquid lubricating coatings increased in the following order: El < UV < Pr < AO (using IL-GO30). As shown in Fig. 4, the ILs and IL-GO30 changed from colorless to brown, and the viscosity increased. The wear scars can be seen. The resulting ILs could not flow back to the wear marks in the friction process and thus lost their lubrication function. Figure 3 shows optical microscopic images of the wear scars of steel films before and after irradiation. Figure 3i,j show an obvious wear track with large width and depth formed on the stainless-steel surface after the wear test. Many grooves along the sliding direction were observed on the wear track, which are attributed to abrasive wear in the friction process.

**Figure 5:**
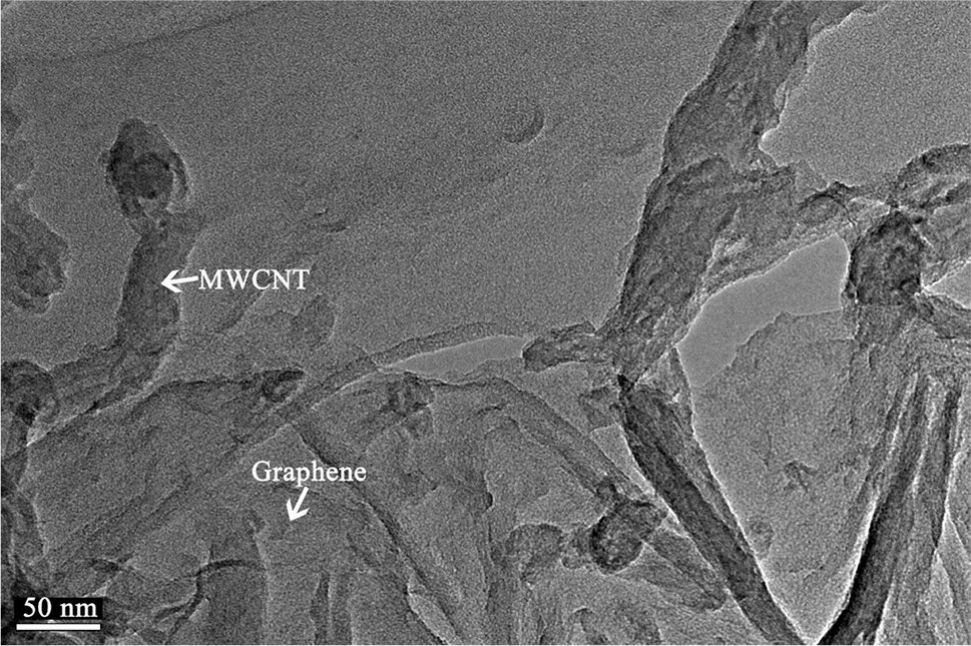
TEM micrographs of the MWCNT and graphene sheets after UV irradiation

Firstly, we separately irradiate the carbon nanomaterials with ultraviolet light; we observe their morphology through TEM. In Fig. 5, we can see that UV radiation's carbon nanomaterials are less affected. Their structure has not changed, and they remain lamellar or tubular. This is consistent with previous literature reports^27^. According to reports in the literature, carbon nanomaterials are less affected by space radiation, their structure has not changed, and they remain lamellar or tubular. Nano-additives can further reduce the friction coefficient and wear rate under the condition of stable ionic liquid. When the ionic liquid changes considerably, the nano-additive loses its effect. Therefore, space irradiation mainly affects ionic liquids to varying degrees.

**Figure 6:**
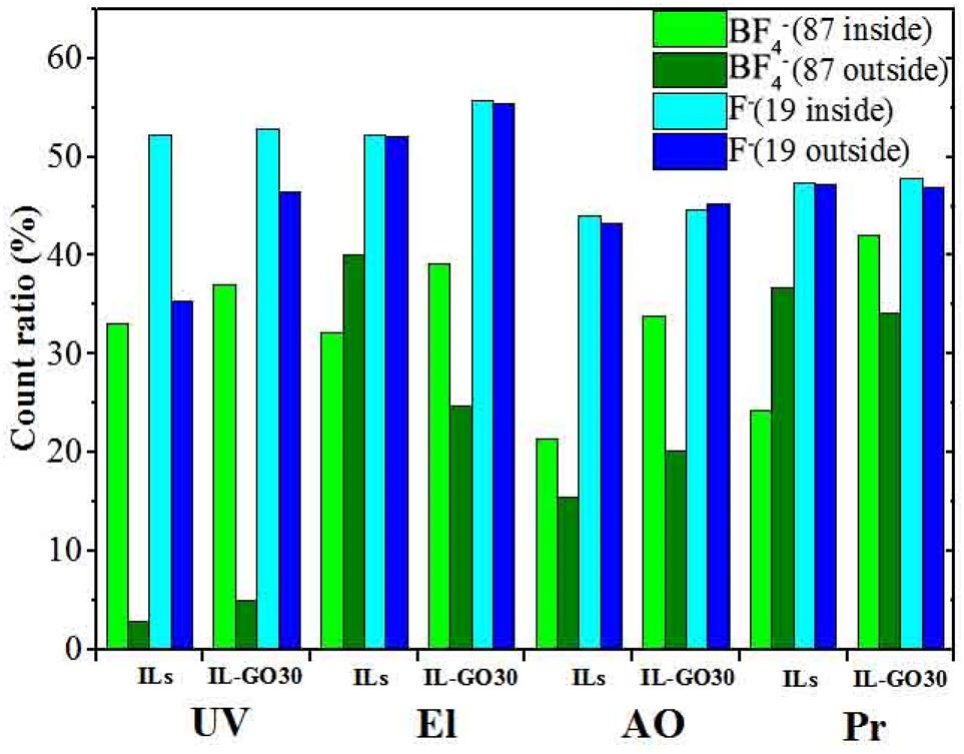
Count ratio obtained via the TOF-SIMS analysis using ILs and IL-GO30.

We studied the wear scars via TOF-SIMS, which is very suitable for surface research. According to previous research, the reduction of the friction coefficient is mainly due to anions reacting with or being adsorbed on the sliding surface^31^. Thus, we only compared the count ratio of anions and F (elements of the anion) inside and outside the sliding track, as shown in Fig. 6. The results showed that the anion and F count ratios inside the sliding track were higher than those measured outside, and several data may be deviation during the test. Such as, for ILs, the count ratios inside the sliding track were lower than those outside under EI and Pr conditions. For IL-GO30, the F count ratio inside was similar to that outside under AO conditions.

**Figure 7:**
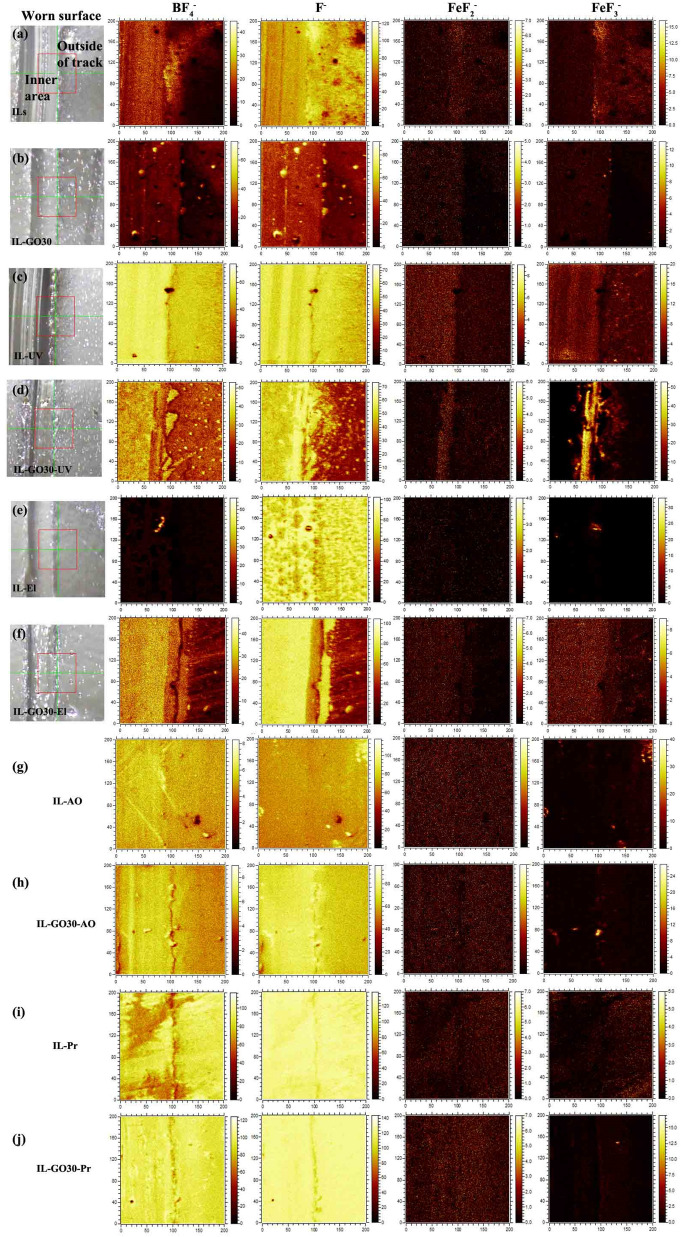
TOF-SIMS image of worn surface lubricated by ILs and IL-GO30 before and after irradiation; from left to right: analyzed area, F^−^, BF_4_^−^, FeF_2_^−^, and FeF_3_^–^.

Imaging TOF-SIMS provided distinct analytical data for the worn surface (Fig. 6). The analyzed area is marked in Fig. 6a. The steel surfaces of ILs and IL-GO30 consist of two typical areas: the "inner area" and the "outer area." Figure 7a–j show chemical images of these analyzed areas. The contents of the elements in the chemical image are expressed by contrast. A bright area indicates a high concentration of the focused element.

After the Pr irradiation, the composite film surfaces had large amounts of BF_4_ and F. Among the four kinds of irradiation, AO and Pr induced the most significant decomposition of the ILs and IL-GO30. The UV-irradiated IL exhibited a small amount of decomposition, which could promote the formation of the tribofilm. Because the F anion reacted with Fe, which was generated by decomposition of the BF_4_ anion and generated Fe−F compounds, this result is consistent with previous experimental results^25,31^. This compound protects the surface of the wear scar. So UV irradiation produces more F anion and has more Fe−F compounds. However, the IL was decomposed to a great extent, and the viscosity of the liquid was significantly increased. In the process of friction, a continuous friction film cannot be formed; thus, the friction coefficient has apparent fluctuations.

**Figure 8:**
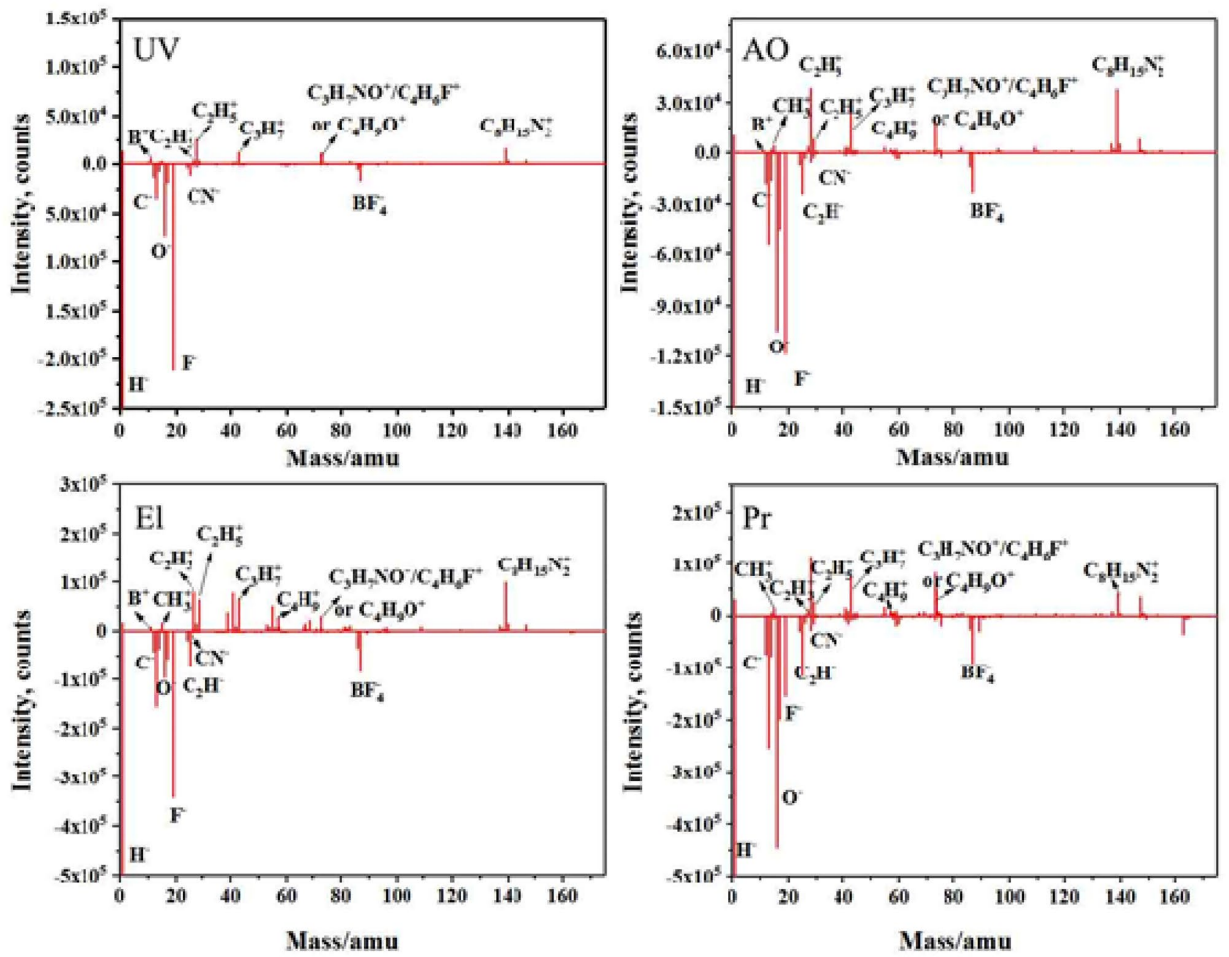
TOF-SIMS spectra of ILs after irradiations.

**Figure 9:**
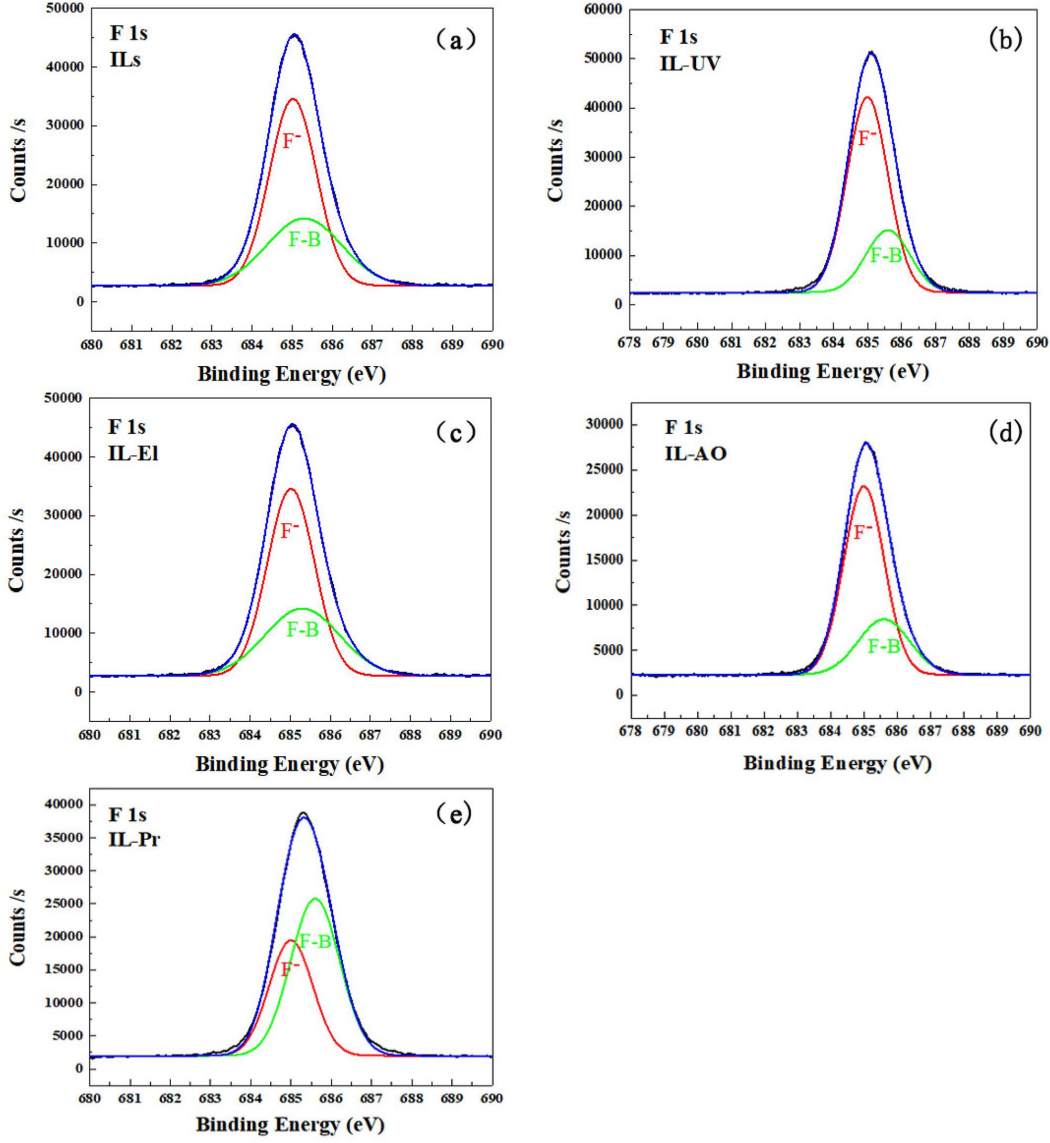
F 1*s* peak in the XPS spectra for ILs before and after irradiation. (**a**) ILs (none irradiation), (**b**) UV irradiation, (**c**) El irradiation, (**d**) AO irradiation, (**e**) Pr irradiation.

According to the experimental results, the ILs were affected by the simulated space irradiation. Thus, the irradiation of ILs without nano additives was performed to compare the degree of IL decomposition. As shown in Fig. 8, the ILs after AO, El, and Pr irradiations showed notable decomposition compared with the case of UV irradiation. Numerous CxHy clusters and other fragments were observed, including B^+^ (m/z = 11 The symbol “m/z” is considered as an abbreviation of the term “mass-to-charge ratio”.), CH_3_^+^ (m/z = 15), C_2_H_3_^+^ (m/z = 27), C_2_H_5_^+^ (m/z = 29), C_3_H_7_^+^ (m/z = 36), C_4_H_9_^+^ (m/z = 57), C_3_H_7_NO^+^/C_4_H_6_F^+^/C_4_H_9_O^+^ (m/z = 73), C_8_H_15_N_2_^+^ (m/z = 139), C^–^ (m/z = 12), O^–^ (m/z = 16), F^–^ (m/z = 19), C_2_H^–^ (m/z = 25), CN^–^ (m/z = 26), and BF_4_^–^ (m/z = 87). After Pr irradiation for 10 min, the ILs molecular structure was significantly degraded and became dry, as confirmed by the X-ray photoelectron spectroscopy (XPS) results in Table 2. As shown in Table 2, the chemical compositions of the IL lubricant changed after the irradiation, especially the C and F elements. The F/C ratios were employed to determine the variations of C and F before and after the irradiations. The F/C ratios of the IL lubricant always decreased after the irradiations. In addition, the obvious changes in F^−^ and F-B content can be seen in Fig. 9. Figure 9 shows the XPS spectrum of F1s, which shows two peaks respectively. The peak appearing at 685.0 eV can be attributed to the peak of F^−^, and F-B appeared at 685.6 ev. After space irradiation, F^–^ obviously increases, and the F^–^/F-B peak area is shown in Table 3. After UV, El, and AO irradiation, the F^−^/ F-B ratio increases, while the proton irradiation F^−^/ F-B ratio is slightly reduced. This indicates that some weak bonds, including the F element in the IL lubricant, were broken during the irradiation, forming small molecular substances and gasified to surrounding circumstances^32^. Therefore, the F/C ratio and F^–^/ F-B ratio decreased after the irradiation, especially after the Pr irradiation.

**Table 2:** The surface compositions (at.%) of ILs after irradiation by XPS.

Surface composition (at%)	Irradiation				No irradiation
	AO	El	UV	Pr	
C	54.8	51.07	48.67	56.24	48.57
N	9.11	10.4	11.6	22.02	12.46
O	7.99	4.87	1.41	3.59	0.44
B	7.3	8.6	9.23	7.99	8.93
F	20.8	25.06	29.09	10.16	29.59
F/C	0.38	0.49	0.60	0.18	0.61

**Table 3:** F^–^/F-B ratios of ILs.

	No irradiation	Irradiation			
		UV	El	AO	Pr
F^–^/F-B	0.66	2.92	1.76	2.67	0.65

### Related wear mechanisms

The results mentioned above propose the mechanism governing the tribological behaviors of the IL/(GO-MWCNT) coatings during the friction process. ILs were most affected by space irradiation, and the effect increased in the following order: UV < El < AO < Pr.

**Figure 10:**
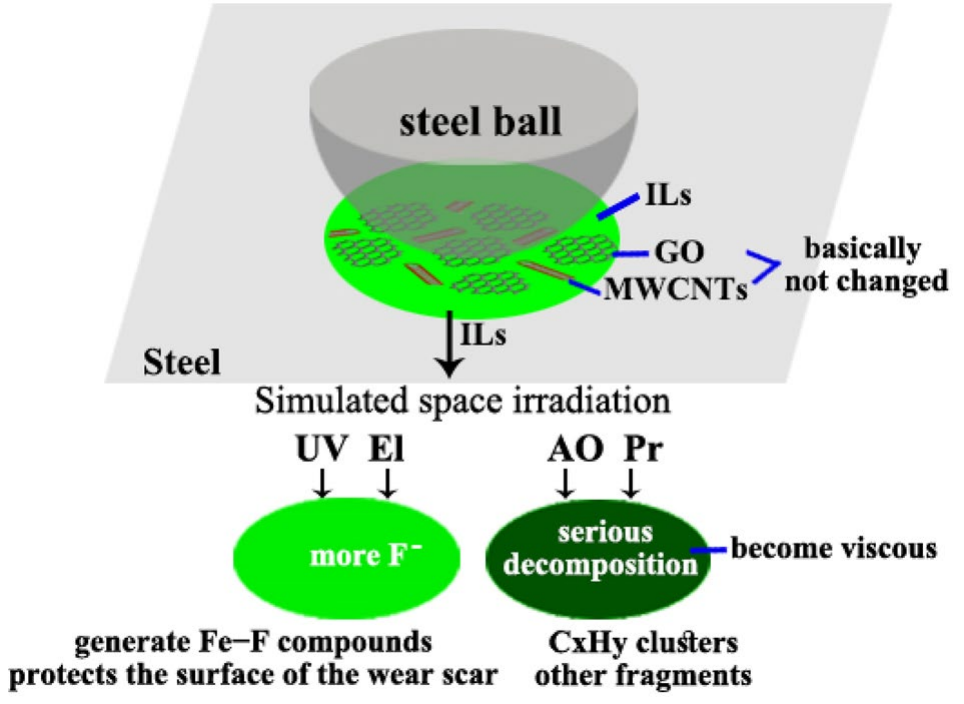
Schematic explaining the possible friction and wear mechanism for the composite coatings after the simulated space irradiation.

The simulated space irradiation induced the degradation of the ILs and the lubricating performance. When a small amount of ILs decompose, the F^-^ produce and react with steel to form a protective film and reduce friction and wear. However, a large number of ILs underwent decomposition and became viscous. Thus, the ILs could not flow back to the wear marks in the friction process and lost their lubrication function. Nano additives reduced the friction and wear in the presence of liquid lubricants. However, the additives were ineffective owing to the considerable decomposition of the IL. Figure 10 shows a schematic of the mechanisms of the composite coatings after the simulated space irradiation.

## Conclusions

The effect of space irradiation on the lubrication performance of IL/(GO-MWCNT) coatings was investigated. The effects of UV, El, AO and Pr irradiations on the tribological and structural properties of ILs were studied in detail. This composite film can effectively resist partial spatial radiation such as UV and El irradiation, leading to more F^–^ forming on the steel surface. These anions reacted with or were adsorbed on the sliding surface, which reduced the friction coefficient and wear rate. AO and Pr irradiation-induced more severe degradation of ILs than UV and El, making the IL/(GO-MWCNT) coatings ineffective during friction. ILs were most affected by the space irradiation. Finding new ionic liquids with carbon nano-materials is essential to tackle these irradiations.

## Data Availability

The datasets used and/or analyzed during the current study are available from the corresponding author on reasonable request.
